# Static Cold Storage with Mitochondria-Targeted Hydrogen Sulfide Donor Improves Renal Graft Function in an Ex Vivo Porcine Model of Controlled Donation-after-Cardiac-Death Kidney Transplantation

**DOI:** 10.3390/ijms241814017

**Published:** 2023-09-13

**Authors:** George J. Dugbartey, Smriti Juriasingani, Mahms Richard-Mohamed, Andrew Rasmussen, Max Levine, Winnie Liu, Aaron Haig, Matthew Whiteman, Jacqueline Arp, Patrick P.W. Luke, Alp Sener

**Affiliations:** 1 Matthew Mailing Center for Translational Transplant Studies, London Health Sciences Center, Western University, London, ON N6A 5C1, Canadajarp3@uwo.ca (J.A.);; 2 Department of Surgery, Division of Urology, London Health Sciences Center, Western University, London, ON N6A 5C1, Canada; 3 Multi-Organ Transplant Program, London Health Sciences Center, Western University, London, ON N6A 5C1, Canada; mahmoud.richard-mohamed@lhsc.on.ca; 4 Physiology & Pharmacology Department, Accra College of Medicine, Accra P.O. Box CT 9828, Ghana; 5 Department of Pharmacology and Toxicology, School of Pharmacy, College of Health Sciences, University of Ghana, Legon, Accra P.O. Box LG43, Ghana; 6 Department of Microbiology & Immunology, Schulich School of Medicine & Dentistry, University of Western Ontario, London, ON N6A 5C1, Canada; 7 Department of Pathology & Laboratory Medicine, Western University, London, ON N6A 5C1, Canada; 8 St. Luke’s Campus, University of Exeter Medical School, Exeter EX1 2HZ, UK

**Keywords:** donation-after-cardiac-death (DCD), ex vivo porcine model, AP39, hydrogen sulfide (H_2_S), ischemia-reperfusion injury (IRI), static cold storage (SCS), kidney transplantation

## Abstract

The global donor kidney shortage crisis has necessitated the use of suboptimal kidneys from donors-after-cardiac-death (DCD). Using an ex vivo porcine model of DCD kidney transplantation, the present study investigates whether the addition of hydrogen sulfide donor, AP39, to University of Wisconsin (UW) solution improves graft quality. Renal pedicles of male pigs were clamped in situ for 30 min and the ureters and arteries were cannulated to mimic DCD. Next, both donor kidneys were nephrectomized and preserved by static cold storage in UW solution with or without AP39 (200 nM) at 4 °C for 4 h followed by reperfusion with stressed autologous blood for 4 h at 37 °C using ex vivo pulsatile perfusion apparatus. Urine and arterial blood samples were collected hourly during reperfusion. After 4 h of reperfusion, kidneys were collected for histopathological analysis. Compared to the UW-only group, UW+AP39 group showed significantly higher pO_2_ (*p* < 0.01) and tissue oxygenation (*p* < 0.05). Also, there were significant increases in urine production and blood flow rate, and reduced levels of urine protein, serum creatinine, blood urea nitrogen, plasma Na^+^ and K^+^, as well as reduced intrarenal resistance in the UW+AP39 group compared to the UW-only group. Histologically, AP39 preserved renal structure by reducing the apoptosis of renal tubular cells and immune cell infiltration. Our finding could lay the foundation for improved graft preservation and reduce the increasingly poor outcomes associated with DCD kidney transplantation.

## 1. Introduction

Kidney transplantation has become the preferred treatment option for patients with end-stage renal disease (ESRD). Although it is not a panacea for kidney disease, it is a superior alternative to long-term hemodialysis, as it confers substantial survival advantage over dialysis therapy and provides quality-of-life benefit at a significantly lower cost to the healthcare system [1,2]. However, there continues to be a global disparity between donor kidney demand and supply due to the increasing number of ESRD patients on the kidney transplant waiting list compared to the number of donor kidneys available for transplantation. This crisis has necessitated an increase in the use of renal grafts from donors-after-cardiac-death (DCD), which has now evolved into routine clinical practice in many countries. Currently, DCD contributes to about 25% of kidney transplantation performed globally, and continues to be on the rise [3,4,5]. Although DCD donors provide a valuable expansion of the pool of donor kidneys for transplantation, these kidneys have an increased rate of delayed graft function (DGF) and primary non-function (PNF), which are important consequences of ischemia-reperfusion injury (IRI) and which together contribute to acute graft rejection and early graft loss, thus negatively impacting graft quality, patient survival, hospital stay and long-term kidney function [6,7,8]. Therefore, there is a growing interest to identify novel strategies to improve the utilization of DCD kidneys and to reduce the associated post-transplant complications.

Hydrogen sulfide (H_2_S), the third established gaseous signaling molecule (gasotransmitter) after nitric oxide (NO) and carbon monoxide (CO), is receiving significant attention together with NO and CO in various experimental models of organ transplantation [9,10,11]. H_2_S has been identified as a potential candidate drug to combat IRI in solid organ transplantation through several molecular mechanisms, such as the inhibition of anti-inflammatory and anti-apoptotic signaling pathways and the activation of antioxidant and vasodilatory mechanisms [12]. Among several compounds that have been investigated as H_2_S donors that minimize IRI in experimental organ transplantation, AP39 [(10-oxo-10-(4-(3-thioxo-3H-1,2-dithiol-5yl)phenoxy)decyl) triphenylphosphonium bromide)] appears to demonstrate a greater potential, as it directly targets the mitochondrion (a major contributor to IRI) and releases H_2_S in a slow and sustained fashion [13]. Considering that experimental kidney transplantation is technically challenging in small animals (e.g., rodents), with anatomical, physiological, and pharmacological differences compared to humans [14,15], we used an ex vivo porcine model, which is not only less challenging, but also attractive due to the anatomical, physiological and body size similarities between pigs and humans. Moreover, porcine kidneys share similar genetics, embryological development, and morphological features as well as similar responses to injury with human kidneys [14,15]. To establish a proof of principle for treatment to ensure that animal models of therapy worked prior to moving on to longer storage times, the present study aims to investigate whether 4 h of static cold storage (SCS) of renal grafts in AP39-supplemented UW solution at 4 °C on ice, which reflects the current clinical standard of care for organ graft preservation, would improve renal graft quality and function in a clinically relevant ex vivo porcine model of DCD kidney transplantation.

## 2. Results

### 2.1. Addition of AP39 to UW Solution Improves Arterial pO_2_ and Tissue Oxygenation during Reperfusion

**Figure 1:**
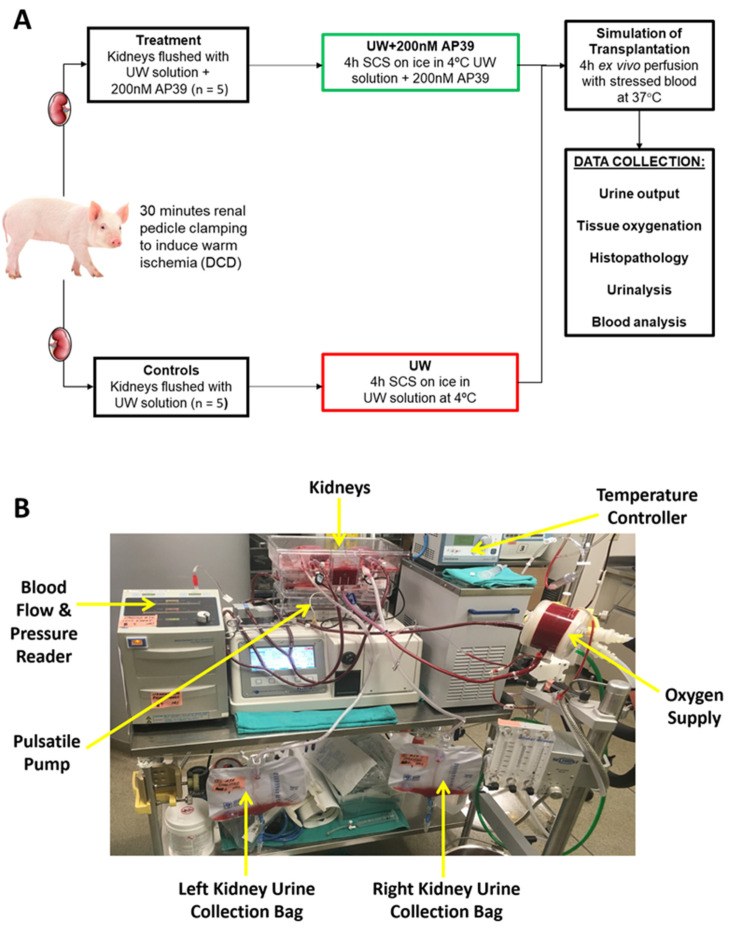
(**A**) Experimental design, showing a summary of the preservation of the groups of renal grafts. (**B**) Ex vivo pulsatile perfusion setup, showing reperfusion after static cold storage. Kidney from each group placed in the perfusion cassette and are receiving stressed autologous blood. Ureters of the kidneys are connected to bags to collect urine and measure the volume of urine produced. A temperature controller is connected to oxygenator to regulate blood temperature.

**Figure 2:**
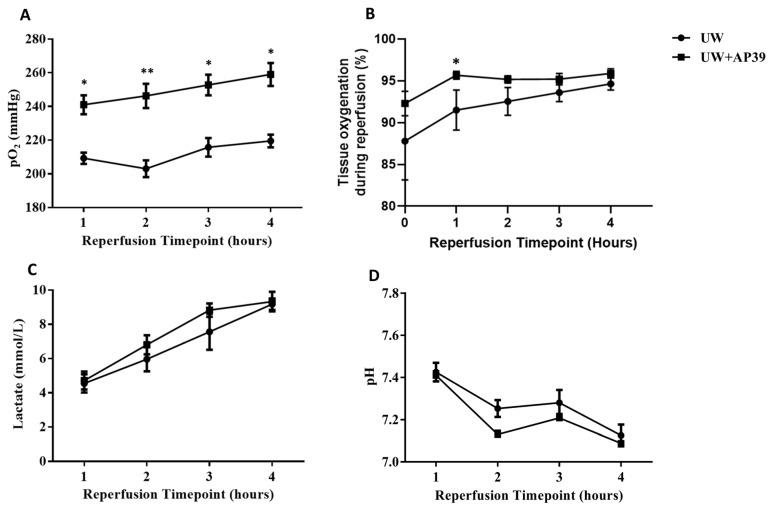
**AP39 improves arterial blood parameters and tissue oxygenation during reperfusion.** (**A**) Arterial oxygen tension (pO_2_) in UW group compared to UW+AP39 group. (**B**) Tissue oxygenation of UW and UW+AP39 kidneys during reperfusion. (**C**) Plasma lactate and (**D**) pH of UW and UW+AP39. Values are mean ± SD. * *p* < 0.01, ** *p* < 0.001.

As illustrated in Figure 1, arterial blood samples were analyzed hourly during reperfusion to evaluate relative changes in oxygen tension (pO_2_), lactate, and pH between the UW-only and UW+AP39 groups. Compared to the UW-only group, the UW+AP39 group showed significantly higher levels of arterial pO_2_ throughout the 4 h reperfusion period, with a much greater difference at the 2 h timepoint (Figure 2A), and was positively correlated with graft tissue oxygenation at the 1 h timepoint (Figure 2B). However, no significant difference was observed in graft tissue oxygenation from the 2 h to the 4 h timepoint, as tissue oxygenation between both groups reached almost the same level with increasing reperfusion time (Figure 2B). Interestingly, the lactate level increased and pH decreased during reperfusion, and there was no significant difference in these parameters between both groups (Figure 2C,D). In summary, the supplementation of the UW solution with 200 nM AP39 improved arterial oxygen tension and renal tissue oxygenation without affecting lactate production and blood pH.

### 2.2. Supplementation of UW Solution with AP39 Improves Renal Graft Function during Reperfusion

**Figure 3:**
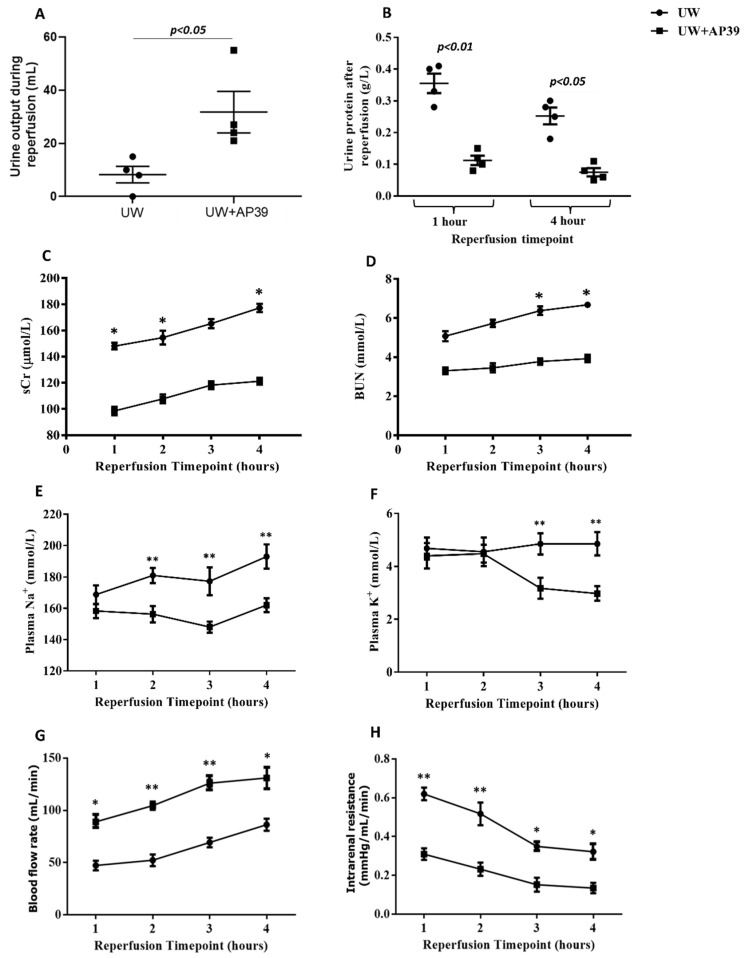
**AP39 improves renal graft function during reperfusion**. (**A**) Urine output between UW and UW+AP39 kidneys during reperfusion. (**B**) Urine protein excreted by UW and UW+AP39 kidneys after reperfusion. (**C**) Serum creatinine between UW and UW+AP39 kidneys; (**D**) blood urea nitrogen between UW and UW+AP39 kidneys; and (**E**) plasma Na^+^, (**F**) plasma K^+^, (**G**) blood flow rate, and (**H**) intrarenal resistance between UW and UW+AP39 kidneys. * *p* < 0.05, ** *p* < 0.01.

To assess the effect of AP39 on renal graft function following its addition to the UW solution, urine and blood samples were collected hourly during reperfusion, and parameters of renal function were measured. The addition of AP39 to the UW solution resulted in a marked increase in urine production compared to the volume of urine produced by the control kidneys without AP39 supplementation (Figure 3A; *p* < 0.05). Since renal graft preservation by static cold storage at 4 °C on ice did not allow for urine collection, urine samples were collected at the 1 h and 4 h timepoints, during reperfusion and urine protein was analyzed between both groups. While urine protein from kidneys of both groups slightly decreased from the 1 h to the 4 h timepoint, urine protein in the UW+AP39 group significantly decreased by over three-fold at the 1 h timepoint compared to that in the UW-only group (Figure 3B; *p* < 0.01), with a similar trend at the 4 h timepoint (Figure 3B; *p* < 0.05). Also, the levels of serum creatinine and blood urea nitrogen in the UW+AP39 group reduced significantly during reperfusion compared to the UW-only group, corresponding to plasma sodium (Na^+^) and potassium (K^+^) levels (Figure 3C–F). In addition, blood flow rate in UW+AP39 group markedly increased throughout reperfusion, with a corresponding decrease in intrarenal resistance compared to the UW-only group (Figure 3G,H; *p* < 0.05). Thus, the addition of AP39 to the UW solution improved renal graft function, as evidenced by increased urine output and blood flow rate, and by reduced levels of urine protein, sCr, BUN, plasma Na^+^ and K^+^, as well as intrarenal resistance.

### 2.3. Storage of Renal Grafts in AP39-Supplemented UW Solution Preserves Graft Structure after Reperfusion

**Figure 4:**
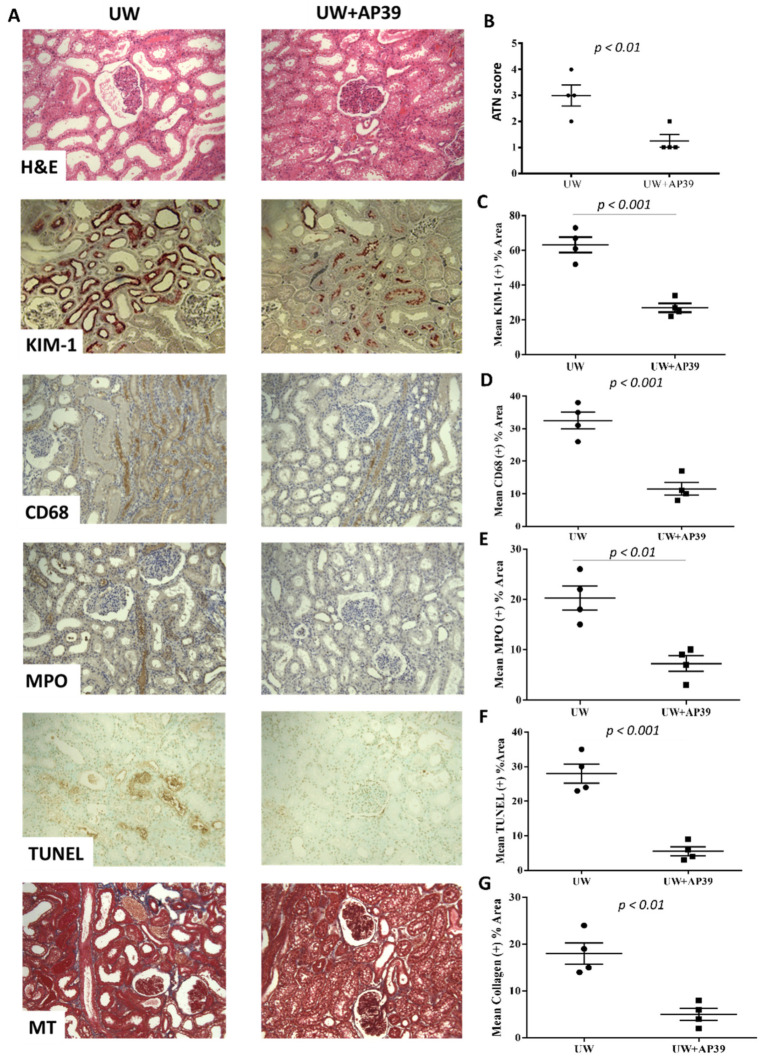
**AP39 preserves renal graft structure after reperfusion**. (**A**) Representative images of UW and UW+AP39 kidneys showing H&E, KIM-1, CD68 (macrophage marker), MPO (neutrophil marker), TUNEL (apoptotic marker) and Masson’s trichrome (fibrotic marker) stainings at 200× magnification. Quantitative analysis of (**B**) acute tubular necrosis score, (**C**) renal KIM-1 expression, (**D**) renal CD68 expression, (**E**) renal MPO expression, (**F**) apoptosis of renal cells, and (**G**) collagen deposition in interstitial compartment. Black dots represent UW only group while black squares represent UW+AP39 group.

To evaluate the impact of AP39 on a renal graft injury, kidney sections from both groups were stained with H&E, KIM-1, CD68, MPO, TUNEL, and Masson’s trichrome. Renal graft morphology, as revealed by the H&E stain, showed a significantly lower acute tubular necrosis score in UW+AP39 kidneys compared to kidneys in the UW-only group (Figure 4A,B; *p* < 0.01). Staining with KIM-1 (kidney injury molecule-1) also showed the same trend (Figure 4A,C; *p* < 0.001). In addition, the influx of macrophages and neutrophils in the kidneys of the UW+AP39 group were markedly lower relative to the UW-only kidneys, as revealed by CD68 and MPO stains, respectively (Figure 4A,D,E). Furthermore, TUNEL and Masson’s trichrome stains also revealed a significant reduction in apoptosis and fibrosis, respectively in AP39-treated kidneys compared to kidneys in the UW-only group (Figure 4A,F,G). Taken together, the preservation of renal grafts in a AP39-supplemented UW solution limits graft injury during static cold storage and reperfusion.

## 3. Discussion

The widening disparity between donor kidney supply and demand has resulted in an increased use of kidneys from DCD donors. Using a clinically applicable ex vivo swine model of controlled DCD kidney transplantation, we have shown in the present study that the supplementation of University of Wisconsin preservation solution with the slow-releasing mitochondria-targeting hydrogen sulfide (H_2_S) donor, AP39, does not only improve arterial oxygen tension and renal graft oxygenation, but also improves graft function, as evidenced by the increased urine output and blood flow rate and decreased levels of urine protein, serum creatinine, and blood urea nitrogen, as well as intrarenal resistance during reperfusion after static cold storage (SCS) on ice at 4 °C. The significant improvement in blood flow rate, with reduced intrarenal resistance and increased arterial oxygen tension and renal graft oxygenation by AP39 suggests a vasodilatory effect of AP39, which facilitated local oxygen delivery and consumption. Our result also showed that AP39 preserved renal graft structure after 4 h of reperfusion, which was characterized by a lower acute tubular necrosis (ATN) score; reduced the renal expression of KIM-1; and decreased graft neutrophil and macrophage infiltration, as well as apoptotic and fibrotic markers without affecting lactate production and blood pH. This finding is consistent with previous rat models, which involved the actual transplantation of donor kidneys and lungs (syngeneic and allogeneic transplantation), where we and others demonstrated graft protection and prolonged recipient survival following 3–24 h of SCS in preservation solution supplemented with AP39 and sodium hydrosulfide (NaHS; another H_2_S donor compound) [16,17,18]. Thus, although SCS on ice at 4 °C is the current clinically approved method of organ graft preservation for transplantation, cold storage is associated with negative graft outcomes due to its contribution to cold ischemia-reperfusion injury (IRI) of the organ graft, with an increased rate of ATN, delayed graft function (DGF), primary non-function (PNF), significantly reduced urine production, and decreased graft survival [19,20,21,22]. Therefore, our findings that SCS in an H_2_S-supplemented preservation solution limits IRI and improves renal graft quality and post-transplant complications are of clinical importance.

Considering that DGF and PNF are important consequences of IRI and associated with DCD kidney transplantation due to prolonged warm ischemic time between cardiac arrest and renal graft procurement, and the fact that mitochondria are a major source of production of reactive oxygen species (ROS; a destructive mediator of cell and tissue injury) and also represent a subcellular target of damage with several consequences during warm ischemia, cold storage, and reperfusion [16,23], it is of interest to protect the mitochondria during organ transplant procedures. This explains why mitochondria are the primary target of H_2_S, as has been demonstrated via the inhibition of the mitoK_ATP_ channel/p38- and JNK-MAPK pathways in several in vitro and in vivo models of IRI, thus highlighting the cytoprotective value of H_2_S [24,25,26]. It is worth noting that while all the complexes of the mitochondrial electron transport chain (ETC) suffer various degrees of structural damage and reduced activity during ischemia, complexes I and III are the most sensitive to ischemic injury [23] and become more susceptible to electron leakage, which continues during and after reperfusion [27]. In addition, prolonged ischemia depletes mitochondrial adenosine triphosphate (ATP) levels and increases anaerobic glucose metabolism, which decreases intracellular pH through increased lactate formation [28,29], and a higher luminal release of intracellular and lysosomal enzymes in DCD kidneys [30]. Interestingly, however, we did not observe a significant difference in plasma lactate and pH between both groups. This may suggest that the dose and duration of the AP39 treatment may not have been optimal enough to cause a significant change in the glycolytic pathway.

Also, ischemia and reperfusion are recognized paradoxical phenomena that impair mitochondrial antioxidant defenses through the increased production of ROS, leading to oxidative damage in cells and tissues, which plays a significant role in graft IRI, as has been shown through the decreased activities of mitochondrial antioxidant enzymes (e.g., glutathione peroxidase and manganese superoxide dismutase) in experimental transplantations [31,32,33]. While we did not measure tissue or plasma antioxidant levels in the present study, it is well-established that the H_2_S released from its donor compounds including AP39, is a potent antioxidant that protects renal and other solid organ grafts from ROS-induced oxidative damage during ischemia and reperfusion in animal models of organ transplantation [34,35,36,37] as well as in other experimental models that closely match the clinical situation of whole-body deep cooling and rewarming [38]. Moreover, we a observed marked reduction in the renal infiltration of neutrophils and macrophages (additional sources of ROS) in the AP39-treated grafts, further suggesting the antioxidant as well as anti-inflammatory effects of H_2_S. The mechanisms through which H_2_S exerts its antioxidant effects include direct ROS scavenging, the modulation of the levels of cytoplasmic and mitochondrial antioxidants (e.g., glutathione peroxidase and thioredoxin), and the activation of the transcription factor nuclear factor (erythroid-derived2)-like2 (Nrf2) [16,39], leading to the increased expression of several antioxidant enzymes that function as a defense net during the process of oxidative stress.

It is important to point out that AP39 is a synthetic compound that directly targets the mitochondria via its triphenylphosphonium motif, which facilitates H_2_S entry into the mitochondria, and has demonstrated a greater potential to improve graft function. Interestingly, among all the H_2_S donor compounds that have been investigated experimentally so far, AP39 makes a significant difference in minimizing IRI through the preservation of mitochondrial integrity, including the restoration of mitochondrial bioenergetics and cellular metabolism by increasing electron transport at complexes I and III (the most sensitive complexes to ischemia) of the mitochondrial ETC under pathological conditions [16,40]. Although the present study did not explore cellular and subcellular mechanisms underlying the renoprotective effect of AP39, it very likely that this mechanistic action of AP39 contributed to the observed renal graft protection. Another factor to consider is the fact that Ca^2+^ regulates oxidative phosphorylation under physiological conditions. However, under stressful conditions, such as ischemia and reperfusion, Ca^2+^ enters the mitochondrial matrix and triggers the opening of mitochondrial permeability transition pores (MPTPs) [41]. Interestingly, AP39 has been reported to prevent the mitochondrial release of pro-apoptotic factors by inhibiting the Ca^2+^-mediated opening of MPTPs and preventing the collapse of the mitochondrial transmembrane electrochemical gradient, thereby protecting against myocardial IRI and other pathological conditions [24,42,43,44]. Bearing this mechanism in mind, one could surmise that AP39 prevents the mitochondrial accumulation of Ca^2+^ during renal graft preservation by SCS since hypothermia promotes the influx of Ca^2+^ and mitochondrial Ca^2+^ overload [45,46,47]. Hence, the addition of AP39 to the UW solution during the SCS of renal grafts in the present study may have prevented MPTP opening and preserved mitochondrial function, leading to protection against IRI and an improvement in renal graft quality and function. The graft-protecting effect of AP39 observed in the present study also supports previous findings in porcine kidneys and murine hearts, in which AP39 was added to the preservation solution and downregulated the expression of pro-inflammatory genes, such as interleukin-6 (IL-6), IL-1β, and tumor necrosis factor-alpha (TNF-α) [13,48]. This suggests that mitochondrial targeting by H_2_S donors, such as AP39 during graft preservation and reperfusion, preserves mitochondrial integrity, thereby contributing significantly to minimizing graft IRI regardless of the preservation technique and temperature, and that AP39 could be a useful pharmacological agent to limit the inevitable problem of IRI and to reduce post-transplant complications.

Another observation in the present study that reflects renal graft dysfunction and injury in DCD kidneys is electrolyte disturbance characterized by the abnormal tubular reabsorption of Na^+^ and K^+^ in the UW group. This result supports that of a recent clinical study involving 76 patients who received DCD kidneys [49]. In their retrospective analysis of plasma electrolytes in the first 3 days after kidney transplant, Hayes et al. [49] reported hyperkalemia in DCD kidney recipients compared to live-donor kidney recipients, which also aligned experimentally with DBD kidneys [30]. Our result in the present study showed that the supplementation of standard preservation solution with AP39 does not only reduce plasma K^+^ levels, but also those of plasma Na^+^. There are studies showing that the pharmacological inhibition of endogenous H_2_S reduces renal blood flow and the glomerular filtration rate, resulting in increased Na^+^ and K^+^ reabsorption, while H_2_S administration through NaHS inhibits the activities of the Na^+^/K^+^-ATPase and Na^+^-K^+^-2Cl^−^ cotransporter in the renal tubules, thereby preventing the reabsorption of these ions and potentiating their excretion [50,51,52]. In greater detail, the inhibitory effect of H_2_S on Na^+^/K^+^-ATPase has been shown to be due to its ability to directly target H_2_S-sensitive disulfide bonds in the epidermal growth factor receptor in the proximal tubule, resulting in endocytosis and the inhibition of Na^+^/K^+^-ATPase via the EGFR/GAB1/PI3K/Akt signaling pathway [52]. In addition, exogenous H_2_S administration prevents the hydrogen-peroxide-induced activation and opening of epithelial sodium channels (ENaC; responsible for sodium reabsorption) in the distal tubule via the phosphatidylinositol 3,4,5-trisphosphate (PI(3,4,5)P3) pathway [53], thereby reducing Na^+^ reabsorption and increasing its excretion. These mechanisms could account for the reduced plasma Na^+^ and K^+^ in the AP39-treated group in the present study.

While the result of the present study holds clinical promise, there are a number of limitations that hamper the translation of our work to clinical studies. First and foremost, our study lacks a healthy control or sham-operated group that could have provided baseline values for comparison. This is due to the high cost that limits the number of groups we could evaluate, as each pig experiment costs over $5000. AP39 is currently not in clinical use, and therefore, its therapeutic efficacy in animal models will need to be translated from bench to bedside. A potential solution is to consider clinically viable H_2_S donors. To this end, our research group is currently investigating the renal-graft-protecting effect of sodium thiosulfate [54], an FDA-approved H_2_S donor drug that is currently in clinical use. In addition, our study did not measure plasma or renal H_2_S levels due to the lack of an effective, valid, and reliable method to measure the endogenous H_2_S concentration in real-time. However, the differential effects between the two experimental groups highlight its impact. In addition, due to limitations in the oxygen saturation machine, we were unable to measure renal graft tissue oxygenation and urine production during SCS, hence we could not compare the oxygenation and urine output of the grafts during SCS or reperfusion. Although this study provides excellent proof of principle, the next steps should be to both preserve the grafts for a longer period of time in SCS to mimic clinical situations. Nonetheless, our short-term results are reassuring and add to the growing body of knowledge about the therapeutic impact of H_2_S. Addressing all these limitations in future studies will shed more light on the therapeutic feasibility of H_2_S in the transplantation of kidney and other transplantable solid organs and will hasten the translation of H_2_S donor compounds from bench to bedside.

## 4. Materials and Methods

### 4.1. Induction of Warm Ischemia to Mimic-Controlled DCD

Male Yorkshire swine (*n* = 5; 60–70 kg) obtained from Caughell Farms, Fingal, Canada, were tranquilized and a midline incision was used to expose the kidneys. Both kidneys with their ureters were dissected free from the retroperitoneum without interfering with the blood supply to the ureter, and then the ureter was divided. The renal artery and vein were freed up to the aorta and vena cava to keep the renal vascular pedicles intact as long as possible [13]. To induce warm ischemia and mimic renal injury due to controlled DCD as well as to facilitate ex vivo perfusion and urine collection, the renal pedicles were clamped in situ for 30 min, and the ureters and arteries were cannulated following the intravenous infusion of 500 units of heparin per kilogram body weight. The complete cessation of renal blood flow replicates the clinical version of DCD, where no oxygen is being supplied to the donor kidneys during procurement. Next, both donor kidneys were nephrectomized. Surgeries were performed by transplant fellows at University Hospital, London, Canada. All experimental procedures were approved by the Animal Use Committee of University of Western Ontario, Canada (Protocol ID: 2018-090).

### 4.2. Preservation of Renal Grafts by Static Cold Storage

Following nephrectomy, the donor kidneys were randomly assigned to the UW group (*n* = 5) or UW+AP39 group (*n* = 5). To mimic the clinical version of SCS, which is the current clinical standard of care for organ graft preservation, paired renal grafts from each pig were flushed with and stored in UW solution (on ice at 4 °C for 4 h with or without AP39 (200 nM)). The UW solution contained water, lactobionic acid, potassium phosphate monobasic, magnesium sulfate heptahydrate, raffinose pentahydrate, adenosine, allopurinol, glutathione, potassium hydroxide, and sodium hydroxide, according to the manufacturer. The experimental design is illustrated in Figure 1A. AP39 was provided by Prof. Matthew Whiteman (University of Exeter Medical School, Exeter, UK), and the dose added to the UW solution was determined from our previous study [13], while the UW solution was obtained from Global Transplant Solutions, Toronto, ON, Canada.

### 4.3. Ex Vivo Perfusion with Stressed Autologous Blood

The ex vivo pulsatile perfusion apparatus used in the present study is illustrated in Figure 1B and identical to the setup used in our previous studies [13]. Following 4 h of SCS at 4 °C, all kidneys were reperfused with stressed autologous blood for 4 h at 37 °C to simulate reperfusion after kidney transplantation. Blood in the perfusion circuit from the donor pig was oxygenated using an external oxygen gas supply, and 5000 units of heparin per 1 L bag was added to prevent clotting [55]. By adjusting the flow, the mean perfusion pressure was maintained at 60 mmHg after an initial 5 min period of gradual increase. Urine and arterial blood samples were collected hourly, and tissue oxygenation (measured with a monitor) and the volume of urine production during reperfusion were recorded. PlasmaLyte solution (Baxter, Deerfield, IL, USA) was used to compensate for the fluid volume loss. In addition, blood parameters (pH, pO_2_, and lactate) were also measured hourly using iSTAT Handheld Blood Analyzer (Abbott Laboratories, Chicago, IL, USA). After 4 h of reperfusion, the kidney samples were collected, cut into sections (including cortex and medulla), and stored in 10% neutral-buffered formalin for histopathological analysis.

### 4.4. Analysis of Renal Function

Following the hourly collection of urine and arterial blood samples, the parameters of renal function, such as serum creatinine, blood urea nitrogen, and plasma electrolytes (Na^+^ and K^+^), were measured using an iSTAT Handheld Blood Analyzer (Abbott Laboratories, Chicago, IL, USA). Urine protein levels were measured using an IDEXX Urine Analyzer (IDEXX Laboratories, Westbook, ME, USA) following a 1:3 dilution of urine in HemogloBind (Biotech Support Group, Monmouth Junction, NJ, USA) to obtain clearer urine samples after 10 min of vigorous shaking and centrifugation at 12,000× *g*. Creatinine values were undetectable in the urine samples.

### 4.5. Histopathological Examination of Renal Grafts

Formalin-fixed kidney sections were embedded in paraffin, cut into 4 μm thick sections for histopathological examination and mounted onto microscope slides. The sections were stained with Hematoxylin and Eosin (H&E) and Terminal deoxynucleotidyl transferase dUTP nick end labeling (TUNEL) to determine the degree of acute tubular necrosis (ATN) and apoptosis, respectively. The stained sections were viewed under a Nikon Instruments Eclipse 90i digital microscope at 200× magnification (Nikon Instruments, Melville, NY, USA). Both sets of slides were scored by a blinded renal pathologist on the basis of staining intensity as per the following previously reported scheme: 1 =< 11%, 2 = 11–24%, 3 = 25–45%, 4 = 46–75%, 5 => 75% [13]. Additional stainings were performed using the following primary antibodies: kidney injury marker (KIM-1; Abcam^®^, Toronto, ON, Canada; diluted 1:500), macrophage surface marker CD68 (Abcam^®^, Toronto, ON, Canada; diluted 1:50), neutrophil-specific enzyme myeloperoxidase (MPO; Abcam^®^, Toronto, ON, Canada; diluted 1:50), and Masson’s trichrome (a marker for tissue fibrosis; Abcam^®^, Toronto, ON, Canada; diluted 1:100) according to the manufacturer’s protocol. The stained sections were viewed under the same microscope at the same magnification and quantified from 20 fields, each using ImageJ software (version 1.8; National Institutes of Health, Bethesda, MD, USA), as we previously reported [49].

### 4.6. Statistical Analysis

All statistical analyses were conducted using GraphPad Prism statistical software (La Jolla, CA, USA; version 9.0). All other data were analyzed using a *t*-test to determine any statistical differences between both groups. Statistical significance was accepted at *p* < 0.05. Values are presented as mean ± standard deviation (SD).

## 5. Conclusions

In conclusion, our findings in the present study provide the first experimental evidence, showing that the supplementation of a conventional organ preservation solution with AP39 during SCS at 4 °C improves renal graft structure and function in a clinically relevant ex vivo porcine model of controlled DCD kidney transplantation. This was evidenced by improved arterial oxygen tension, tissue oxygenation, renal hemodynamics, and preserved renal graft structure and function. Based on our experimental data, our study provides novel insights into a promising future clinical outcome of DCD kidney transplantation following H_2_S supplementation during SCS. Considering that DCD kidneys are associated with reduced viability and characterized by delayed and/or reduced urine production and filtration capacity, as the lack of perfusion due to prolonged warm ischemic time increases tissue damage, our findings in the present study could lay the foundation for improved renal graft preservation and reduce the high incidence of post-transplant complications associated with DCD kidney transplantation. This will meet the rising demand for kidney transplantation, which ultimately will reduce the transplant waiting list, shorten the waiting times, and reduce the morbidity and mortality associated with long-term dialysis therapy.

## Data Availability

The original contributions presented in the study are included in the article.
